# GAB2 couples genetic drivers and signaling networks in acute myeloid leukemia

**DOI:** 10.1172/JCI198684

**Published:** 2025-11-03

**Authors:** Amanda Luvisotto, Lu Wang

**Affiliations:** 1 Department of Biochemistry and Molecular Genetics and; 2 Simpson Querrey Center for Epigenetics, Feinberg School of Medicine, Northwestern University, Chicago, Illinois, USA.

## Abstract

In acute myeloid leukemia (AML), leukemogenesis is typically driven by the sequential acquisition of distinct classes of mutations that collaborate to transform normal hematopoietic stem and progenitor cells. The founding and cooperating mutations in AML are often in signaling genes and form functional partnerships with each other, each addressing complementary aspects of malignant transformation. In this issue of the *JCI*, Kramer et al. elaborate on the molecular pathogenesis of AML. By using a mouse bone marrow model bearing the common AML-initiating mutations in DNA methyltransferase 3 α (DNMT3A) and nucleophosmin 1 (NPM1), the work provides further evidence for the role of the signaling orchestrator GRB2-associated–binding protein 2 (GAB2) in AML progression, positioning GAB2 as a potential therapeutic target.

## The mutational landscape of acute myeloid leukemia

Acute myeloid leukemia (AML) is a bone marrow malignancy that originates from myeloid progenitor cells through uncontrolled clonal proliferation (1). By the end of 2025, over 22,000 new cases of AML will be diagnosed, and more than 11,000 patients will die from the disease (2). The standard therapeutic regimen consists of combination chemotherapy with an anthracycline and cytarabine, a protocol that has been used for over 50 years (3–5). Recently, the approval of targeted therapies on an individual basis has reached clinical practice and broadened the range of treatment options for patients (4, 6). However, despite these advancements, relapses are common, and refractory disease is frequently observed. AML remains highly aggressive, with a dismal 5-year survival rate of only 32% (2, 6, 7).

Myeloid cells are important components of immunity, as they play multiple roles in supporting the immune system (8, 9). Nevertheless, when their proliferation and differentiation become dysregulated, these cells can become the origin of illness (10). Genetic abnormalities are common in AML and play an important role in leukemogenesis, although the complete pathophysiology of the disease has not yet been fully clarified (11, 12). After initiation by concurrent, cooperating mutations, AML pathogenesis is supported by the accumulation of alterations that confer survival advantages to myeloid cells (13). Some common driver mutations in AML occur in the genes DNA methyltransferase 3 α (*DNMT3A*), nucleophosmin 1 (*NPM1*), fms-like tyrosine kinase 3 (*FLT3*), ten-eleven translocation methylcytosine dioxygenase 2 (*TET2*), *TP53*, and isocitrate dehydrogenase (*IDH*) (10–15). These alterations often act synergistically to contribute to AML progression, and the diversity of mutations underscores the heterogeneity of this disease (11, 12). Moreover, chromosomal structural rearrangements are also frequent in AML, particularly the t(15;17) translocation, which results in the PML::RARA fusion protein and is strongly associated with acute promyelocytic leukemia (APL), a subtype of AML (16).

Malignant cells are often transformed at an early myeloid stage in AML (10, 17), and molecular characterization of AML provides important insights into diagnosis, prognosis, and treatment strategies (4, 5, 18). Understanding how the molecular landscape of AML contributes to disease progression is crucial for uncovering new precision medicine approaches aimed at improving patient outcomes.

## Chromosome amplification identified in *Dnmt3a*^R878H/+^ *Npm1*^cA/+^ mutant animal models

**Figure 1:**
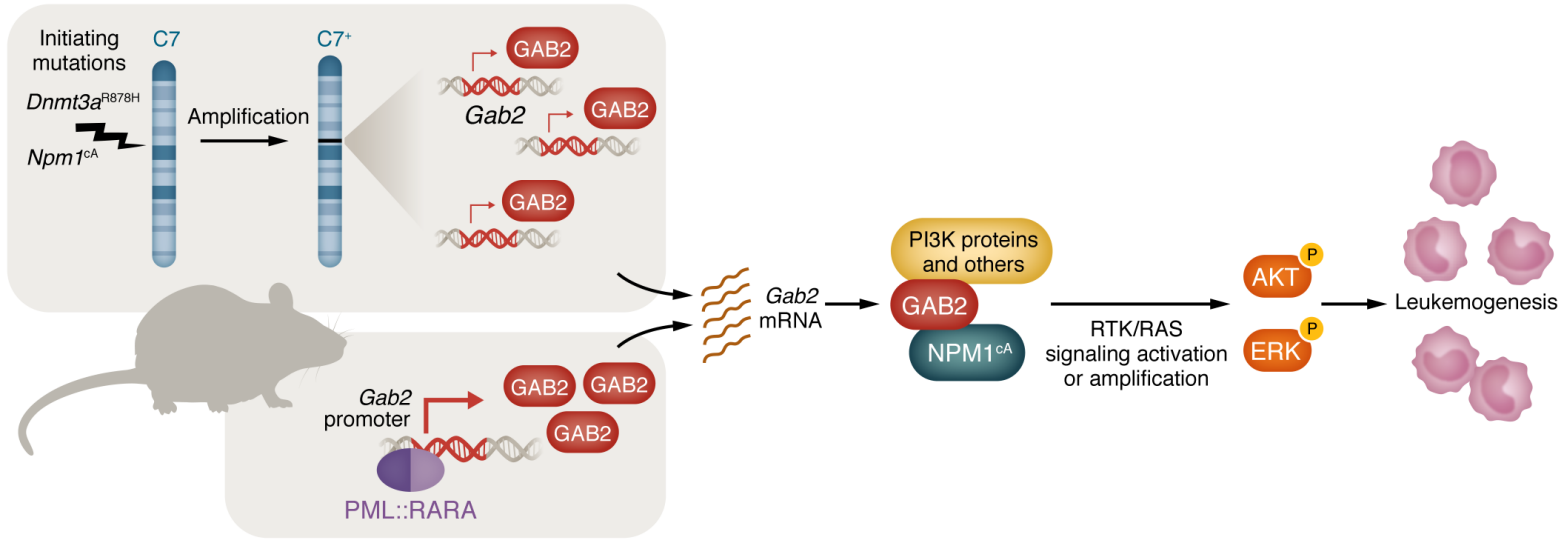
GAB2 links initiating mutations and progression signaling in myeloid leukemogenesis. AMLs arising in a mouse model expressing two common initiating mutations (*Dnmt3a*^R878H^ and *Npm1*^cA^) frequently acquire a single-copy amplification of chromosome 7, which includes the *Gab2* gene locus. Overexpression of *Gab2*, driven by genetic amplification and/or the PML:RARA fusion protein, can accelerate AML development and promote the survival of fully transformed AML cells. The unique GAB2 interactome formed in the presence of mutant NPM1 activates downstream AKT and ERK signaling in a posttranscriptional manner, highlighting a critical role for GAB2 as a potentially targetable “missing link” between AML-initiating mutations and RTK/RAS pathway mutations associated with AML progression.

The *DNMT3A* and *NPM1* genes are among the most frequently mutated in AML, each affecting 20%–30% of patients (10, 19, 20). These mutations co-occur more often than expected by chance, with approximately 15% of patients with AML harboring alterations in both genes (15). Previous studies have also identified additional cooperating factors in patients with *DNMT3A* and *NPM1* mutations, such as mutations in the RTK/RAS signaling pathway (21). In the present study, Kramer and colleagues sought to elucidate the mechanistic links between the initiating mutations in *DNMT3A* and *NPM1* and facilitating mutations within the RTK/RAS signaling pathway (22). The researchers used a well-established mouse model carrying a heterogeneous germline *Dnmt3a*^R878H/+^ mutation and a conditional *Npm1*^cA/+^ allele, together with a tamoxifen-inducible (TAM-inducible) flippase transgene to activate the *Npm1* mutation (23). Once myelomonocytic AML had developed in these mice, the team performed whole-genome sequencing and identified human AML–like cooperating mutations, including alterations in *Ptpn11*, *Kit*, *Cbl*, and *Nf1*. Additionally, they observed amplification of murine chromosome 7 in nearly all samples (10 of 11) (Figure 1). Notably, similar amplifications were identified in two nonleukemic mice in this study, supporting the interpretation that this amplification may be an intermediate step during AML progression.

## GAB2 facilitates AML progression induced by *Dnmt3a*^R878H/+^ *Npm1*^cA/+^ mutations

Next, Kramer et al. set out to determine which of the 206 genes within the minimally amplified region on chromosome 7 was responsible for bridging the gap between initiating mutations and subsequent facilitating mutations or activation of RTK/RAS pathway components (22). Using RNA-Seq analysis of AML cells derived from the mouse model, they narrowed their focus to two genes, GRB2-associated–binding protein 2 (*Gab2*) and p21-activated kinase 1 (*Pak1*) (Figure 1), which were also identified as highly essential in AML based on the DepMap analysis (24). In the subsequent validation, Kramer and colleagues found that retroviral expression of *Gab2*, but not *Pak1*, markedly stimulated clonal expansion in *Dnmt3a* *Npm1* double-mutant cells in vivo. Interestingly, *Gab2* overexpression conferred a selective disadvantage in both wild-type and *Dnmt3a*-mutant bone marrow, whereas the combination of *Npm1* and *Dnmt3a* mutations synergized to create a permissive environment favoring *Gab2* overexpression. Additional cooperating mutations — such as in *Nf1*, *Ep300*, and *Notch1* — were observed in *Gab2*-overexpressing AMLs, with only one case showing mutations in RTK/RAS signaling genes. This suggests that *Gab2* overexpression may, at least in part, reduce the requirement for additional RTK/RAS activation.

## The unexpected role of GAB2 in posttranscriptional regulation in AML

To further investigate the underlying mechanism, Kramer et al. performed bulk RNA-Seq analysis to identify potential changes in transcriptional programs between GFP-expressing and *Gab2*-overexpressing cells isolated from mice at different time points (22). Surprisingly, no significant differences in gene expression were detected between *Gab2*-overexpressing and GFP-expressing cells. Although GAB2 may also influence cellular function by regulating mRNA splicing, the researchers chose to focus on the GAB2 protein interactome in *Dnmt3a Npm1* mutant cells. To this end, they used the TurboID system to label intracellular proteins in close proximity to GAB2 as well as to either wild-type or mutant forms of DNMT3A and NPM1 in primary hematopoietic stem and progenitor cells (HSPCs). Notably, they observed a substantially increased interaction between GAB2 and mutant NPM1 in cells carrying the *Dnmt3a*^R878H/+^ mutation, but not in cells with wild-type *Dnmt3a*. This finding suggests that GAB2 may modulate cellular function via posttranscriptional mechanisms.

To further validate this hypothesis, the team conducted global tandem mass tag (TMT) proteomics analysis alongside bulk RNA-Seq and identified several key RTK/RAS signaling proteins, including GRB2, MAPK1, and PIK3CD, that were elevated at the protein level in bone marrow of mice with myelomonocytic AML without corresponding increases in mRNA levels. Consistent with this result, the phosphorylation levels of AKT (Ser473) and ERK (Thr202/Tyr204) were markedly increased in GAB2-expressing myelomonocytic AML cells (Figure 1).

## GAB2 dependency in murine and human AML cells

Next, the researchers sought to determine whether *Gab2* is required for the viability of fully transformed murine AML cells. As expected, depletion of *Gab2* via CRISPR markedly reduced cell viability in in vitro culture, underscoring its strong relevance to human AML pathogenesis and highlighting its potential as a therapeutic target. Kramer et al. then examined *GAB2* mRNA levels in primary human AML samples from The Cancer Genome Atlas (TCGA) database and found that GAB2 expression was higher in PML::RARA-positive tumor samples than in normal CD34^+^ cells (22). Consistently, DepMap analysis revealed a marked dependency on GAB2 in a subset of human leukemia cell lines compared with nonmyeloid cancer cells (24). Notably, the patient-derived AML samples harboring mutant NPM1 and FLT3-ITD fusions were more sensitive to GAB2 depletion than normal human CD34^+^ HSPCs from cord blood.

## Transcriptional activation of *Gab2* by the PML::RARA fusion protein

Finally, given the elevated expression of GAB2 in PML::RARA-positive primary AML, the researchers investigated whether the PML::RARA fusion protein could act as a transcriptional regulator of GAB2 in human and mouse AML cells. Indeed, they observed substantial occupancy of the PML::RARA fusion protein at the *GAB2* promoter regions, which was abolished upon treatment with all-*trans* retinoic acid (ATRA) — a compound that is known to degrade the PML::RARA protein (25, 26). Single-cell RNA-Seq analysis in mice expressing the PML::RARA fusion protein in *Ctsg*-expressing myeloid progenitors further revealed a dramatic increase in *Gab2*^+^ cells within a unique population of myeloid precursor cells that is found exclusively in the bone marrow. These findings suggest a potential therapeutic approach in which targeting the PML::RARA fusion protein using small-molecule inhibitors or degraders such as ATRA could block the ability of the fusion protein to activate GAB2 expression.

## Final remarks and future perspectives

The findings presented by Kramer et al. advance our understanding of the complex molecular landscape of AML progression, highlighting GAB2 as a key signaling coordinator in the context of DNMT3A and NPM1 mutations (19, 20, 27, 28). Moreover, the data suggest that GAB2 upregulation is promoted by the PML::RARA fusion protein, thereby intensifying its expression in both mouse and human samples. This work identifies GAB2 as a potential missing link between a driving mutation event in AML and downstream signaling, positioning it as a potentially relevant therapeutic target.

While this study presents a well-conceived design and yields interesting findings, several key questions remain to be addressed in future explorations. For instance, despite insights from the GAB2 interactome analysis, a further understanding of how GAB2 interacts with mutant NPM1 and how the GAB2/NPM1^cA^ activates protein stability of factors within RTK/RAS signaling will greatly extend our knowledge of the role of GAB2 in leukemogenesis. Ultimately, these insights may affect the development of diagnostic strategies and targeted therapies, enhancing patient prognosis and survival.

## Funding support

This work is the result of NIH funding, in whole or in part, and is subject to the NIH Public Access Policy. Through acceptance of this federal funding, the NIH has been given a right to make the work publicly available in PubMed Central.

- NIH (R35GM146979, to LW).
- American Cancer Society, Research Scholar Grant (RSG-22-039-01-DMC).
- United States Army Medical Research Acquisition Activity (USAMRAA) Idea Development Award (HT94252310360).

## Version 1 11/03/2025

Electronic publication
